# Investigation of a simple suspension method for orexin receptor antagonist tablets

**DOI:** 10.1186/s40780-026-00553-7

**Published:** 2026-02-14

**Authors:** Kazuhiro Hada, Daichi Inoue, Kouyou Ohishi, Toshiya Yasunaga, Kayoko Ozeki, Hiromitsu Yamamoto, Nobuhiko Nakamura

**Affiliations:** 1 Laboratory of Pharmacy Practice and Science, School of Pharmacy, Aichi Gakuin University, Nagoya, Japan; 2 Medical System Network Co., Ltd., Sapporo, Japan; 3 Laboratory of Pharmaceutical Engineering, School of Pharmacy, Aichi Gakuin University, Nagoya, Japan

**Keywords:** Simple suspension method, Orexin receptor antagonist, Belsomra, Dayvigo, Quviviq

## Abstract

**Background:**

A simple suspension method is used in medical practice to administer several orexin receptor antagonists for patients with dysphagia; however, detailed suspension and passage through catheter evaluations have not been performed. Therefore, to safely administer orexin receptor antagonists using the simple suspension method in patients with dysphagia, this study evaluated the disintegration, suspension, passage through the catheter, and drug recovery after passage of Belsomra^®^ 20 mg (suvorexant), Dayvigo^®^ 10 mg (lemborexant), and Quviviq^®^ 50 mg (daridorexant) tablets through a catheter.

**Methods:**

Disintegration and suspension of Belsomra^®^ 20 mg, Dayvigo^®^ 10 mg, and Quviviq^®^ 50 mg tablets, and passage through the catheter were visually confirmed. Drug recovery after passage through the catheter was quantified using liquid chromatography-mass spectrometry.

**Results:**

In this study, there was no disintegration and suspension of Belsomra^®^ 20 mg tablets. However, Dayvigo^®^ 10 mg and Quviviq^®^ 50 mg tablets demonstrated disintegration and suspension, including passage through the catheter after rapid and complete disintegration. Drug recovery after catheter passage was >80% for Dayvigo^®^ 10 mg without flushing and for Quviviq^®^ 50 mg after two flushes.

**Conclusion:**

As the disintegration and suspension properties of Belsomra^®^ 20 mg tablet could not be confirmed, the conventional simple suspension method is not suitable for this product. However, Dayvigo^®^ 10 mg and Quviviq^®^ 50 mg tablets showed potential suitability for the simple suspension method.

**Supplementary Information:**

The online version contains supplementary material available at 10.1186/s40780-026-00553-7.

## Background

The number of patients with dysphagia has increased in Japan owing to the increasing population of older adults [1]. Dysphagia has various causes, and stroke, cognitive impairment, trauma, and loss of swallowing function associated with aging are also contributing factors [2–5]. Many of these patients take several medications to treat their illnesses, and some are unable to continue treatment because of swallowing difficulties [6].

Medications are available in various dosage forms, including tablets, capsules, injections, and topical medicines. Oral administration is simple and non-invasive, and patients can administer it themselves. Most drugs used in the medical field are in tablet form [7], and as this is difficult for patients with dysphagia, pharmacists make these tablets in a powder form according to doctors’ instructions. However, powdering and decapsulating tablets increase pharmacist’s workload and cause problems such as drug loss [8].

The simple suspension method is gaining attention as a solution to these challenges. In the simple suspension method, tablets and capsules are disintegrated and suspended in warm water without tablet powdering or decapsulation. The suspended tablets are then administered via syringe or through the nose tube, gastrostomy, or enterostomy [9, 10]. This method of administration is mainly indicated for older adults and patients with dysphagia, and compared with the conventional powdering method, it has less effect on physicochemical stability, simplifies dispensing operations, and alleviates problems such as drug loss.

Many older adults have sleep disorders [11], and approximately 14.5% of individuals aged ≥65 years use sleeping pills [12]. Orexin receptor antagonists induce sleep by antagonizing the binding of orexin to orexin receptors in the brain, and are used clinically to treat insomnia. Barbiturates, histamine receptor antagonists, benzodiazepine receptor agonists, non-benzodiazepine receptor agonists, and melatonin receptor agonists have been used to treat insomnia. However, orexin receptor antagonists are now recommended for older adults because they are more effective at inducing sleep and have fewer side effects, including next-day carryover effects. There are three orexin receptor antagonists on the Japanese market: Belsomra, Deyvigo, and Quviviq, which have different affinities for orexin receptors 1 and 2 [13–15]. These medications are only available in tablet form, but for patients with swallowing difficulties, they must be administered in crushed form or using a simple suspension method. However, since the types and compositions of each additive differ, there may be variations in stability and suspension properties. Dayvigo tablets have been administered using the simple suspension method in some institutions; however, this is not recommended by pharmaceutical companies’ Q&A (faq-medical. http://eisai.jp/faq/show/15098?category_id=997). For Belsomra tablets, the drug information form does not include disintegration, suspension properties, or passage through a catheter. Therefore, the use of simple Belsomra suspension methods is not recommended. Additionally, the suitability of the simple suspension method for Quviviq tablets is unknown; this is because the product has only been available on the market for a short time.

A more detailed study of the simple suspension method is needed to ensure the safe use of orexin receptor antagonists in clinical settings. Therefore, this study aimed to determine the suitability of the simple suspension method for Belsomra^®^ 20 mg, Dayvigo^®^ 10 mg, and Quviviq^®^ 50 mg tablets for patients with dysphagia. This was achieved by confirming disintegration, suspension, passage, and recovery of the drugs as they passes through a catheter.

## Methods

### Materials

Belsomra^®^ 20 mg (MSD, NJ, USA), Dayvigo^®^ 10 mg (Eisai, Tokyo, Japan), and Quviviq^®^ 50 mg (Shionogi, Osaka, Japan) tablets, injection syringe (20 mL: Terumo, Tokyo, Japan), Luer cap (Terumo), injection needle (18 G: Terumo), and Jayfeed nutritional catheter (Bis[2-ethylhexyl] phthalate-free polyvinyl chloride, 8 Fr, 120 cm: JMS, Tokyo, Japan) were used in this experiment.

### Simple suspension method

The simple suspension method was used as with minor modifications previously described [9, 10, 16]. Previous reports state that tablets should be placed in water at 55 °C and left for 5–10 min. However, in the present study, the tablets were left in water for 10 min. One tablet of Belsomra^®^ 20 mg, Dayvigo^®^ 10 mg, or Quviviq^®^ 50 mg was taken and put into a syringe. Additionally, 20 mL of warm water at 55 °C was aspirated into the syringe and left for 10 min after fitting the Luer cap. The syringe was inverted and mixed 15 times. If disintegration and suspension could not be confirmed, a scratch of several millimeters was made on the tablet. The tablet was then retested. After disintegration and suspension, the suspension was passed through a catheter placed 30 cm above the ground at a rate of approximately 20 mL/10 s. The catheter was flushed twice (flush-1 and flush-2) with water (20 mL).

### Assessment

Disintegration was assessed visually by observing breakdown of the tablet coating agent and disintegration of the tablet after leaving it in water at 55 °C for 10 min. The suspension was visually evaluated by confirming that the tablets had lost their shape after being inverted and mixed 15 times and that the particles were dispersed uniformly. Passage through the catheter was assessed by visually confirming that the suspension had passed through the catheter without obstruction [17]. Drug recovery after passage through the catheter was assessed using liquid chromatography-mass spectrometry (LC-MS, 8040; Shimadzu, Kyoto, Japan). Samples were taken after confirmation of tablet disintegration and suspension, after passage through the catheter, after the flush-1 and flush-2 of water at 55 °C, diluted 100,000-fold with acetonitrile, and then estazolam-*d5* (internal control) was added (final concentration was 10 ng/mL) and measured.

### LC-MS/MS analysis

Experiments that used LC-MS/MS to measure the concentrations of suvorexant, lemborexant, and estazolam-*d5* were based on previous reports [18–22]. Measurement conditions for daridorexant were added under similar conditions. Suvorexant, lemborexant, and daridorexant were determined in positive ion mode using an LC system (Nexera 2; Shimadzu, Kyoto, Japan) connected to a triple quadrupole mass spectrometer (LC-MS 8040; Shimadzu) with an electrospray ion source. LC separation of suvorexant, lemborexant, daridorexant, and estazolam-*d5* was performed using a C18 column (2.1 × 50 mm, 3 μm particle size; GL Sciences, Tokyo, Japan) maintained at 35 °C. Separation was performed using gradient elution with mobile phase A (0.1% formic acid in water; mobile phase A) and B (0.1% formic acid in acetonitrile; mobile phase B [MPB]). The gradient program was run as follows: 40% MPB (linear gradient, v/v) at 0 min, 40–90% MPB at 4 min, 90% MPB at 2 min, and 90–40% MPB at 2 min at a flow rate of 0.2 mL/min with a run time of 8 min for each 3 μL injection volume. Multiple reaction monitoring mode was applied for the detection of suvorexant, lemborexant, daridorexant, and estazolam-*d5* by monitoring the transitions: *m/z* 451.25 to 186.1 for suvorexant, *m/z* 411.25 to 287.05 for lemborexant, *m/z* 451.25 to 202.05 for daridorexant, and *m/z* 300.15 to 272.1 for estazolam-*d5*. Details of the LC-MS/MS conditions used for these measurements are provided in Table 1.

**Table 1:** Summary of optimized parameters for LC-MS/MS analysis

Parameters	Suvorexant	Lemborexant	Daridorexant	Estazolam-*d5*
Column	C18 column (2.1 × 50 mm, 3 μm particle size)			
MPA	0.1% formic acid in water			
MPB	0.1% formic acid in acetonitrile			
Linear gradient	40% MPB for 0 min			
	40–90% MPB at 4 min			
	90% MPB at 2 min			
	90–40% MPB at 2 min			
Flow rate	0.2 mL/min			
Injection volume	3 μL			
Column temperature	35 °C			
Polarity	Positive ESI			
Molecular Weight	450.93 g/mol	410.42 g/mol	450.93 g/mol	299.77 g/mol
Precursor (*m/z*)	451.25	411.25	451.15	300.15
Product (*m/z*)	186.10	287.05	202.05	272.10
Q1 pre-bias (V)	−16	−14	−11	−20
CE (V)	−24	−16	−31	−26
Q3 pre-bias (V)	−20	−30	−21	−28
Detection time (min)	4.226	3.045	2.53	2.087
Interface voltage (kV)	Depends on tuning the file	Depends on tuning the file	Depends on tuning the file	Depends on tuning the file
Nebulizing gas (L/min)	3	3	3	3
DL temperature (°C)	250	250	250	250
Heat block temperature (°C)	400	400	400	400
Drying gas (L/min)	15	15	15	15

MPA, mobile phase A; MPB, mobile phase B

### Statistical analysis

All data are presented as mean ± standard error of the mean. Statistical analyses were performed using GraphPad Prism 6 software (GraphPad Software, San Diego, CA, USA). Statistical significance was determined using one-way analysis of variance, and the Tukey–Kramer multiple comparison test was used for post-hoc analysis when *F* ratios were significant. All the data and results of the statistical analyses are presented in Additional file 1.

## Results

### Disintegration

**Fig. 1:**
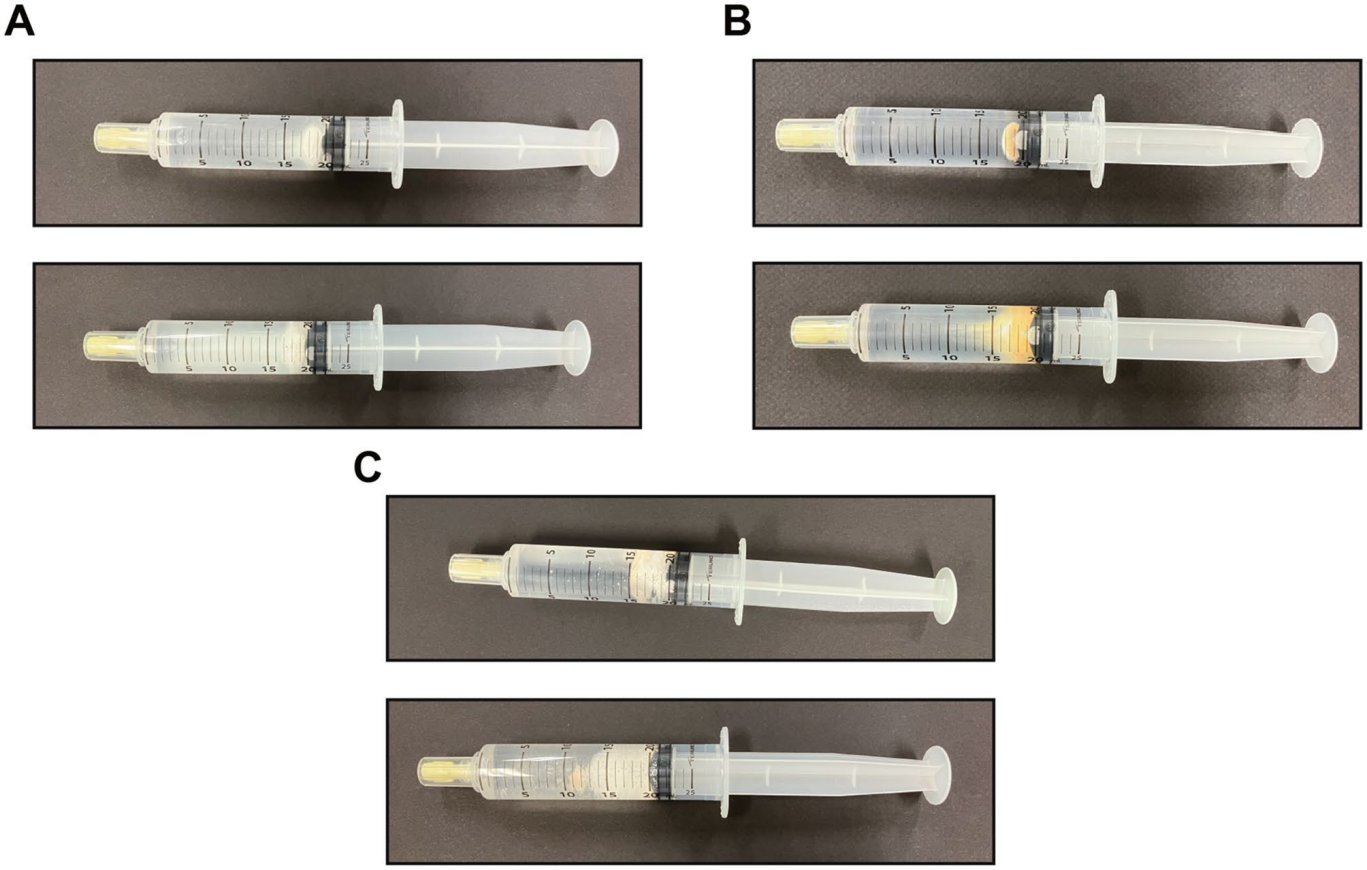
Disintegration of Belsomra^®^ 20 mg, Dayvigo^®^ 10 mg, and Quviviq^®^ 50 mg tablets. Representative images showing Belsomra^®^ 20 mg (**A**), Dayvigo^®^ 10 mg (**B**), and Quviviq^®^ 50 mg (**C**) tablets before and after being left in water at 55 °C for 10 min. The image above shows the tablet after it was inserted, and the image below shows the tablet after 10 min

Disintegration was evaluated by placing Belsomra^®^ 20 mg, Dayvigo^®^ 10 mg, or Quviviq^®^ 50 mg tablets in a syringe containing 20 mL of water at 55 °C and left for 10 min. Subsequently, disintegration was visually confirmed. Belsomra^®^ 20 mg tablet partially disintegrated, and it retained the shape of the tablet (Fig. 1A). However, Dayvigo^®^ 10 mg tablet dissolved completely within 5 min (Fig. 1B). Additionally, Quviviq^®^ 50 mg tablet dissolved completely as soon as it was left in water (Fig. 1C).

### Suspension

Suspension was evaluated after confirming disintegration, and the syringe containing the tablet was inverted and mixed 15 times. The suspension was then confirmed visually. Belsomra^®^ 20 mg tablets were only partially suspended, and they retained their tablet shape (Supplemental Fig. 1A). However, Dayvigo^®^ 10 mg and Quviviq^®^ 50 mg tablets were completely suspended (Supplemental Figures 1B and C). Next, Belsomra^®^ 20 mg tablets were lightly tapped with a pestle to create cracks in them, and the resulting suspension was examined using the same procedure. Belsomra^®^ 20 mg tablets were not completely suspended, with some remaining in their original shape (Supplemental Fig. 1D).

### Passage through the catheter and drug recovery

**Fig. 2:**
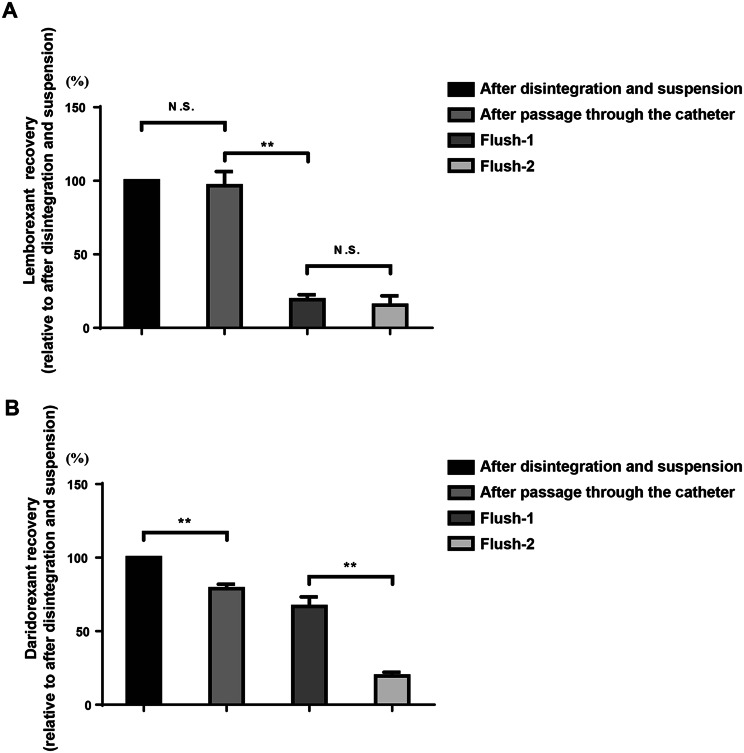
Drug recovery of lemborexant and daridorexant. (**A**) lemborexant recovery after passage through the catheter, and following the flush-1 and flush-2 with water at 55 °C. ** *p* < 0.01. All data are expressed as means ± *SEM* (*n* = 6 samples for each group). (**B**) daridorexant recovery after passage through the catheter, and following the flush-1 and flush-2 with water at 55 °C. ** *p* < 0.01. All data are expressed as incremental recovery and as means ± *SEM* (*n* = 5 samples for each group). *SEM*, standard error of the mean

Passage through the catheter was evaluated after confirming the suspension, which was injected into the catheter. Passage through the catheter was visually confirmed. However, since the suspension characteristics of Belsomra^®^ 20 mg tablets could only be partially confirmed, an assessment of passage through the catheter was not performed. As a result, both Dayvigo^®^ 10 mg and Quviviq^®^ 50 mg tablets passed through the catheter without obstruction. Furthermore, the catheter was washed twice with 55 °C water, allowing remaining particles in the catheter to pass through (Supplemental Fig. 2A, B, C, and D). Drug recovery was measured using LC-MS/MS. Suspension samples were collected after tablet disintegration and suspension, after passage through the catheter, and after flush-1 and flush-2. The results showed that drug recovery of lemborexant was similar after passage through the catheter (*F* [3, 20] = 60.87, *p* < 0.01; Fig. 2A). In contrast, a significant difference in daridorexant recovery was observed after passing through the catheter (*F* [3, 16] = 82.28, *p* < 0.01; Fig. 2B). However, > 80% of the drug passed through the catheter after flush-2.

## Discussion

This study examined three types of orexin receptor antagonists (Belsomra^®^ 20 mg, Dayvigo^®^ 10 mg, and Quviviq^®^ 50 mg tablets) using the simple suspension method. Belsomra^®^ 20 mg tablet is unsuitable for the simple suspension method, while Dayvigo^®^ 10 mg and Quviviq^®^ 50 mg tablets showed potential suitability.

To evaluate disintegration, one tablet of each drug was placed in 20 mL of water at 55 °C and left for 10 min. Dayvigo^®^ 10 mg and Quviviq^®^ 50 mg tablets showed good disintegration, but Belsomra^®^ 20 mg tablet hardly disintegrated. Disintegration of tablets is generally determined by the type and amount of disintegrants, porosity and size of the pores within the tablets, tableting pressure, tablet hardness, presence or absence of a waterproof lubricant, and differences in lubrication methods. Additionally, these drugs contain disintegrants (Belsomra^®^ 20 mg, croscarmellose sodium [CCS]; Dayvigo^®^ 10 mg tablet, low-substituted hydroxypropyl cellulose [L-HPC]; and Quviviq^®^ 50 mg tablet; CCS). Both Belsomra^®^ 20 mg and Quviviq^®^ 50 mg tablets contain CCS. CCS is a superdisintegrant because of its excellent disintegration properties [23, 24]. However, Quviviq^®^ 50 mg tablet disintegrated, while Belsomra^®^ 20 mg tablet did not. After waiting 10 min, film coating of Belsomra^®^ 20 mg tablet peeled off, but the tablet itself did not disintegrate. One possible reason for this is that Belsomra^®^ 20 mg tablet contain copovidone. Suvorexant is insoluble in water; however, it is dispersed at the molecular level in copovidone as a solid dispersion [25, 26]. Copovidone is a hydrophilic polymer that forms a viscous solution when dissolved in water (https://www.pmda.go.jp/files/000229700.pdf). We considered that the viscosity of copovidone slowed water penetration, thereby preventing tablet disintegration. Additionally, Quviviq^®^ 50 mg tablet contains D-mannitol. D-mannitol is a highly soluble excipient. It is used in orally disintegrating tablets because it disintegrates rapidly [27, 28]. Dayvigo^®^ 10 mg tablet has good disintegration properties, which are thought to result from the content of L-HPC as a disintegrant. L-HPC is insoluble in water. It absorbs water and increases water volume. This increase in volume causes tablets to disintegrate quickly, ensuring better and faster tablet disintegration [29]. However, the type and amount of disintegrants, porosity, and size of the pores within the tablets, tableting pressure, tablet hardness, presence or absence of a waterproof lubricant, and differences in lubrication methods are unknown. Therefore, it is difficult to evaluate disintegration solely based on the type of disintegrant used.

After being inverted and mixed, coating of Belsomra^®^ 20 mg tablets dissolved, and the tablets disintegrated slightly while maintaining their shape, but did not completely disintegrate or suspend. Therefore, it was not possible to accurately evaluate the suspension properties of the Belsomra^®^ 20 mg tablets. In contrast, Dayvigo^®^ 10 mg and Quviviq^®^ 50 mg tablets exhibited suspension properties because these characteristics were favorable.

Evaluation of the catheter passage of Dayvigo^®^ 10 mg and Quviviq^®^ 50 mg tablets showed that they passed through the catheter without obstruction. This indicates their suitability for administration via nasogastric, gastrostomy, or enterostomy catheters. Evaluation of drug recovery for lemborexant showed no significant difference after passage through the catheter, suggesting that the residue in the catheter is also low. In contrast, daridorexant significantly decreased the drug recovery after passage through the catheter without flushing.

Both the adhesion/precipitation of the suspension onto the catheter and adsorption of drug molecules onto the catheter can be considered factors influencing drug recovery after catheter passage. Although adsorption would make it difficult to remove a drug by flushing, most of the drug was detected after second passage, suggesting that adsorption was minimal. The adhesiveness and precipitation of the suspension are influenced by various factors such as particle size, zeta potential, viscosity, pH of the suspension, and presence or absence of thickening agents. In the case of Quviviq^®^ 50 mg tablet, the inclusion of crystalline cellulose with a relatively large particle size, as well as the possible presence of a comparatively high amount of povidone, which increases viscosity, may have contributed to the adhesion and precipitation of the suspension within the catheter. Supplemental Figure 2A shows the residual drug after the first passage. Accordingly, in the case of Quviviq^®^ 50 mg tablet, it is considered that the precipitates were flushed out during the flush-2, enabling the recovery of > 80% of the drug.

This study has several limitations. First, evaluations of disintegration, suspension, and catheter passage primarily rely on visual inspection. Second, this study did not examine drug pharmacokinetics after suspension. Further investigation is needed to determine how changes in formulation characteristics are caused by crushing and suspension. Third, although drug recovery rates have been evaluated, the efficacy of drugs obtained using this method has not been evaluated. Therefore, it is necessary to verify the efficacy of these drugs when administered using a simple suspension method. Fourth, this study was based on formulations and specifications available in Japan. For formulations with the same active ingredients used in other countries, differences in the formulation shape or excipients may lead to different results, requiring international validation.

## Conclusions

In this study, Belsomra^®^ 20 mg tablet clarified the causes of unsuitability for the simple suspension method. Additionally, measuring drug recovery after passage through the catheter using LC-MS/MS allowed for a more reliable evaluation than visual evaluation. Consequently, Dayvigo^®^ 10 mg and Quviviq^®^ 50 mg tablets showed potential suitability for the simple suspension method.

Further detailed investigation will enable the provision of safer, evidence-based medical care for patients with dysphagia.

## Electronic supplementary material

Below is the link to the electronic supplementary material.

Supplementary material 1

Supplementary material 2

## Data Availability

All data used in this study are included in the text and supplemental materials.

## Abbreviations

CCS: Croscarmellose sodium
LC-MS/MS: Liquid chromatography tandem mass spectrometry
L-HPC: low-Substituted hydroxypropyl cellulose
MPB: Mobile phases B
