# Supplementary figures and images for: Investigation of a simple suspension method for orexin receptor antagonist tablets
Source: J Pharm Health Care Sci. 2026 Feb 14;12:36. doi: 10.1186/s40780-026-00553-7 (PMC13011777; doi:10.1186/s40780-026-00553-7)

Supplemental figure 1.

**A**

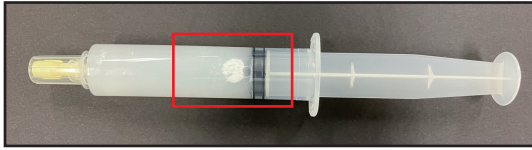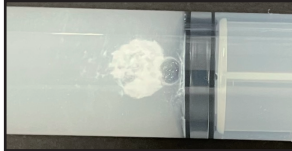

**B**

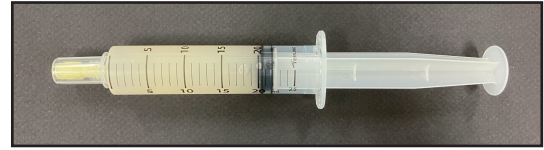

**C**

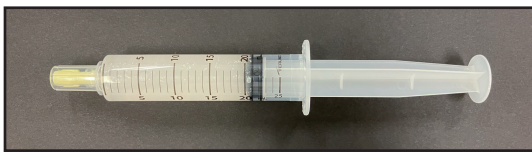

**D**

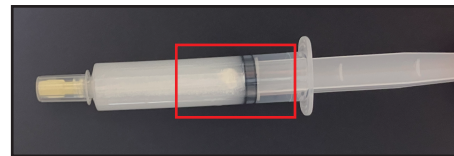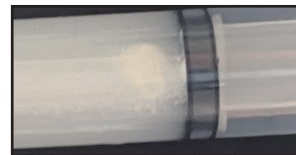

**A**

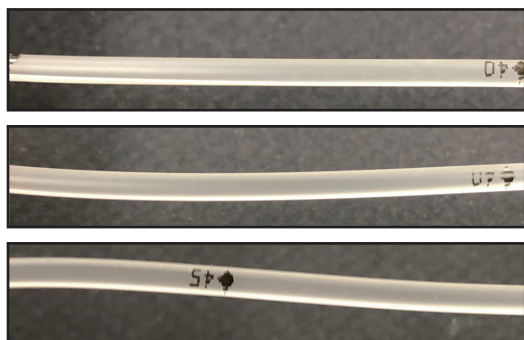

**B**

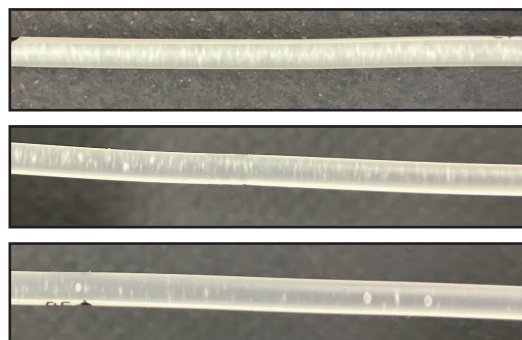

**C**

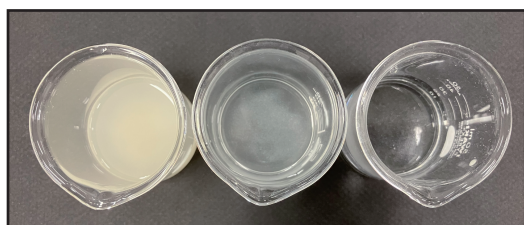

**D**

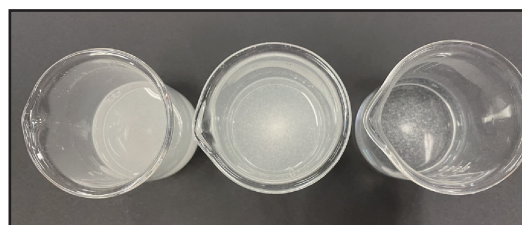

Supplement: Supplementary file 2 — Supplementary material 2 [file 40780_2026_553_MOESM2_ESM.pdf]
